# Some Facts in Pathological Anatomy Regarding the State of the Spleen in the Intermittent Fever of Madagascar

**Published:** 1852-08

**Authors:** 


*Some facts in Pathological Anatomy regarding the state of the spleen in the intermittent fever of Madagascar.* By Dr. Rochard.—Dr. Rochard had an opportunity of observing a large number of severe cases of intermittent fever in Madagascar, in 1830. He gives the following particulars regarding the state of the spleen.

In 153 carefully performed necropsies, he found the spleen diminished in 31 cases; in 22 of these, the patient had not taken the smallest quantity of sulphate of quinine. In the remaining 9, there was nothing abnormal in the state of the spleen, before quinine was given. In 122 patients who took sulphate of quinine in average and daily doses of a gramme (15½ grains) for several days, the spleen always became greatly enlarged: and the same happened with some who had taken no quinine. The enlarged and softened organ exhaled an odour resembling that proceeding from the marshes.

From his observations, Dr. Rochard concludes that—1. The spleen is not the starting point of intermittent fevers. Like all internal organs, it may be congested: and very often its volume, instead of being enlarged, is notably diminished.

2. The frequent increase in size of the spleen is due to its spongy tissue permitting it to become more gorged with blood, under the influence of febrile congestion.

3. Sulphate of quinine does not always diminish the size of a congested spleen.

4. The individual varieties which the size of the spleen presents in man or in animals, in health or in disease, prevents us from appreciating in an exact manner the influence of sulphate of quinine or chloride of sodium in diminishing its volume.

5. Antiperiodic remedies arrest the febrile attacks, not by diminishing the size of the spleen, but by modifying, in a special manner, the economy in general. This modification brings about a state of reactionary equillibrium, under which the miasmatic influence is eliminated from the body.—*Ibid.*

